# Dental Lesions and Their Relation to Nasal and Accessory Cavities

**Published:** 1899-10

**Authors:** G. L. S. Jameson

**Affiliations:** Philadelphia


### International Dental Journal.

Vol. XX. October, 1899. No. 10

### Original Communications.[1]

### DENTAL LESIONS AND THEIR RELATION TO NASAL AND ACCESSORY CAVITIES.[2]

BY G. L. S. JAMESON, D.D.S., PHILADELPHIA.

Owing to the great progress that has been made in the science of medicine during the last decade, it is impossible for one to thoroughly master and practise every branch of this great science. More and more it is being realized that, in order to obtain the highest results, the individual must devote his efforts to some special part after he has acquired a general knowledge of the body as a whole, and in particular of the parts connected more or less directly with his chosen field of practice. Thus it is that certain specialists, other than stomatologists, should have a thorough knowledge of the dental organs, and the converse of this is equally true of the dentist, for frequently in dental lesions the systemic condition of the individual plays a most important part. It is a well-known clinical fact that certain diatheses, especially the gouty and rheumatic conditions, and those associated with various chronic lesions of the internal organs, as the kidney and liver, together with the pathological alterations of the blood, affect directly secreting

[1] The editor and publishers are not responsible for the views of authors of papers published in this department, nor for any claim to novelty, or otherwise, that may be made by them. No papers will be received for this department that have appeared in any other journal published in the country.

[2] Read before the Academy of Stomatology, Philadelphia, April 25, 1899.

surfaces, and that there is frequently deposited on or in the tissues solid materials which act as irritants. The mucous membrane, which is a secreting surface, also aids in the elimination of such irritating substances. It is also true that there is a tendency for this material to be deposited about joints or bony articulations. Now, while the teeth in their relation to the alveolar process of the maxillæ are not exactly a joint or bony articulation, yet they are histologically identical, and at the junction of the tooth with the alveolar process such deposit may take place. Furthermore, in anæmic conditions, where generally faulty nutrition exists, the first part to suffer is that near the joints or articulations; for this reason,—that the higher the grade of tissue the greater is the demand for nutrition, and when nutrition fails, these high-grade structures are the first to suffer. Now, many lesions of the teeth are directly associated with and dependent upon such etiological factors. It is in these conditions that the dentist should have a thorough knowledge of general medicine.

The important relation that the two specialties—stomatology and rhinology—bear to each other is clearly demonstrated by the fact that the entire superior maxilla, both as to its formation and structure, is largely dependent upon and controlled by nasal lesions in early childhood. Any irregularity in breathing due to nasal obstructions in early life, while the facial bones are flexible and of partially cartilaginous union, controls the contour and facial expression. Any condition lessening nasal breathing, in addition to changing the facial expression, will also bring about irregularly formed accessory cavities, especially the antrum. Through failure of the proper amount of air to pass through the nostrils, the facial bones are pressed upon largely by the air in one direction only,—that is, from without. In order to supply the lungs with sufficient air the patient breathes, particularly during sleep, with the mouth open, subjecting the superior maxilla to air-pressure from the exterior, and to muscular pressure also, contracting the arch and producing marked irregularity of the teeth, as well as giving the characteristic droop to the nose. This is produced by the action of the muscles of the face. This muscular action also causes narrowing of the nasal orifice, and although the intranasal obstruction be removed later in life, this abnormally formed orifice will, acting as a valve on inspiration, close the nostril and interfere with nasal breathing, frequently causing in adult life confirmed mouth-breathing.

As in early childhood the teeth are influenced by nasal conditions, so in later life is the nose more or less affected by pathological conditions of the dental organs. Recent clinical observations by stomatologists, rhinologists, pathologists, aurists, and neurologists have clearly proved that many lesions of the head and face, the cause of which has been more or less obscure, have their origin in abnormal or pathological conditions of the dental organs, and at the present time many physicians are beginning to realize the important *rôle* played by the teeth, directly and indirectly, in producing lesions, especially the neuroses, and are acquiring a better knowledge of the too long neglected field of the stomatologist. Already several medical schools have established chairs on diseases of the teeth and oral cavities, and it would be well if all colleges conferring the degree of M.D. should follow their good example.

Of all the specialists of medicine next to the stomatologist, no one requires so thorough a knowledge of the dental organs as the rhinologist; since the teeth are by their position intimately related, directly and indirectly, with the nasal cavity. Before entering upon the diagnosis and treatment of pathological conditions of the teeth affecting the nose and accessory cavities, it would be in order to describe minutely the anatomy of the parts involved, but as so much has been done recently by members of our profession, you are referred to the writings of such men as Drs. Cryer, Garretson, and Marshall. For the best anatomical description of the parts considered in this article, you are referred to a paper read by Dr. M. H. Cryer before the American Dental Association, August 8, 1895, and published in the *Dental Cosmos* of January, 1896; and also to "Studies of Maxillary Bones," read by the same author before the Dental Society of the State of New York, May 11 and 12, 1898, and published in the *Dental Cosmos* of November, same year. These papers have been most favorably commented upon not only by the dental but by the medical profession for their excellent description of the bones and sinuses of the face individually and correlatively, and it is through such articles by stomatologists that the medical profession is awakening to the fact that an important part is played by the dental organs, when abnormal or in a pathological condition, in producing reflex neuroses, nasal and other lesions. Dr. Cryer in his original anatomical researches, by sections and cross-sections, has given the medical profession facts that heretofore have been largely speculative.

The abnormal and pathological conditions of the teeth superinducing or causing nasal obstructions and disturbances may be studied under the following divisions: (1) Teeth inverted and irrupting into the nasal cavity; (2) teeth whose roots are separated from the floor of the nasal or antral cavity by a thin lamina of bone, and which when affected by chronic pericementitis or phagedenic pericementitis, cause inflammation in the floor of the nose by contiguity of tissue. The pulp-chambers of such teeth when devitalized produce alveolar abscess, which breaks through the line of least resistance, and discharges into the nose or antrum. However, if the roots of the teeth are separated from the *antrum* by a thin lamina of bone or even penetrate the sinus, and are covered only by the muco-periosteum, should the teeth become infected, will cause in the antrum empyema. If no infection occurs, there will be evolved gas from tissue-decomposition, giving rise to emphysema or ozæna.

There are certain cases, not here classified, which come under the notice of the stomatologist and rhinologist, where one or more of the incisor teeth, which are separated by a thin lamina of bone from the floor of the nose, become affected with pericementitis, or phagedenic pericementitis. In several cases which have come under my notice it has been a peculiar fact that the tooth affected was the one in direct line with the nasal cavity, in which there was some obstruction close to the floor of the nose, and directly over the root of the diseased tooth. As to the theories as to why these particular teeth should be affected, the most plausible one is that the tooth extending close to the floor of the nose, the root predisposed, becomes inflamed from the collection of secretion and necessary irritation within the nasal cavity, possibly aggravated by some constitutional condition, such as a uric acid diathesis. This chronic inflammation affecting the root would unquestionably force down that tooth slightly, thus bringing the point more into prominence, and subjecting it to greater irritation. Now, if a pathological condition be present, such as uric acid diathesis with deposit, the tooth subjected to this irritation would be more likely to become diseased, which would explain the isolated cases of pericementitis or pyorrhœa alveolaris. Or, granting that there was no systemic condition, but the intranasal obstruction existed, and this obstruction was so located that the accumulation of secretion came directly over the root of the tooth, separated only by a thin lamina of bone, the inflammatory process is set up, causing prominence of that tooth, subjecting it again to more irritation from friction, there is formed at the root of the tooth, as a result of this chronic irritation, new connective tissue, which, following the law of all new inflammatory connective tissue, must contract. This contraction in itself will keep up the pressure on the tooth equal to, if not greater than, the original inflammatory pressure. The constant strain on this tooth will bring about an inflammation, pericementitis, and, with infection following the line of least resistance, very soon a sinus will form and you will have that condition so commonly seen by the stomatologist. The following cases illustrate this condition.

Case I.—Miss W., aged eighteen years. A young lady in the best of health. The teeth were in good condition and the gums healthy, except the left upper central, which was somewhat elongated and slightly separated from the lateral. There was no apparent cause for the condition of this tooth. The tooth continued to elongate, and in spite of all treatment developed into a decided case of phagedenic pericementitis, and the tooth will undoubtedly have to be extracted and possibly replanted. The patient has had nasal irritation for a number of years, particularly in the left nostril, and has had several operations. My attention was called to the condition of the septum, involving the base of the left nostril, by Dr. D. Braden Kyle, who suggested that a spur or redundancy on the septum, under which the secretions accumulated, was the irritating cause of the diseased condition of the tooth. The redundant tissue of the septum projected to almost the floor of the nose, permitting only the passing of a thin probe beneath. The secretions accumulated at this point and formed crusts. The entire nostril was inflamed. The alveolar process was very short and undoubtedly the root of the tooth was very near the floor of the nose. As every other tooth was free from any sign of pericemental irritation, it seems quite certain that the inflammation in the floor of the nose by contiguity of tissue was, as pointed out by Dr. Kyle, the exciting cause of phagedenic pericementitis.

Case II.—Mr. G., aged thirty-five years. The teeth and gums were in fairly good condition. The right upper central was elongated and pushed forward. There was discharge of pus from the gums around the root. A probe could be passed to the apex of the root, showing the peridental membrane separated from about one-quarter circumference of the root.

The nerve was devitalized and pulp removed. The tooth was cut off at the gum-margin, and the natural crown, which had been removed, was ground to occupy a proper position and dowelled to the root. After two years the tooth is free from any discharge, and has only slightly elongated since operation. As this was the only tooth affected with pyorrhœa, I had the patient's nose examined by a rhinologist, and a spur was found on the septum about one-eighth inch from the floor of the nose, directly over the affected tooth. The patient said that he had had for years more or less trouble and irritation of that nostril.

FIRST DIVISION.

*Teeth inverted and irrupting into the Nasal Cavity.*

Case I. (Reported by Professor C. N. Peirce.)—Boy, aged eighteen years. Left superior central incisor missing, and in its place a prominence somewhat resembling a hard tumor. The gum was lanced and alveolus cut away, when the curved root of a tooth was exposed. With considerable difficulty the tooth was extracted by means of forceps, grasping the hooked or curved root presenting. On removal of the tooth an opening was left communicating with the floor of the nose. In this case no particular inconvenience had been given the patient, and only the prominence over the place of central incisor led to interference. In order for this tooth to irrupt into the nose, crown presenting, the dental germ must have been rotated 180 degrees.

Case II. (Reported by Zuckerkandl.)—It has been observed that incisor teeth sometimes develop in the floor of the nose, their crowns pushing up into the anterior part of the nasal fossa. This anomaly is only possible by supposing a rotation of 180 degrees of the dental germ. The enamel, instead of growing downward, directs itself towards the nasal fossa. In this instance the tooth is lengthened and penetrates the nasal fossa. Salter, quoted by Sternberg, has seen a dental germ completely inverted in such a manner that the crown was in place of the root. These inverted teeth were always superior incisors, according to Salter. One could see the crown in the nostrils, from which one is obliged to extract the tooth.

Case III.—Roy, of the Sooree Charitable Hospital, India, reports a case of a Hindoo boy, aged fourteen years, who presented himself for treatment for what was considered a tumor growing in his nose. For four months he had a fetid catarrh with occasional epistaxis. For the previous two months the growth had been observed occupying the left nasal cavity, where it seemed to be attached to its wall at its upper part, its free end looking downward in the shape of a truncated cone.

The tumor was seized with dressing forceps and extracted. It proved to be a canine tooth. The free extremity was covered with enamel, which stopped short at its junction with the root. The root was deeply embedded in the side and upper part of the antrum. The boy had got his set of permanent teeth, with canines and incisors on both sides. There was no deformity of the jaw and no swelling or cystic formation. It was clearly a case of extrafollicular development, an irruption of a tooth in the wrong place, the peculiarity being that, while in reported cases of a like nature the crown of the tooth shows itself at the floor of the nasal cavity, from below upward, in the present instance the dental follicle was transposed, and the irruption was from above downward. The tooth is in the English Royal College of Surgeons' Museum.

SECOND DIVISION.

Case I.—Dr. Alexander W. MacCoy, Philadelphia, reports the following: Anna S., of New Jersey; white; aged forty-five years, came under my care, having the following history: Family history shows phthisis and eczema; personal history has always been delicate. Shortly after birth she had a gathering just below the right orbit, which opened and remained a suppurating sore for several years. There was extensive necrosis of the bones of the face on that side. This finally healed after the condition had lasted for several years. The depression resulting from the loss of bone can be seen and felt. She was born at full term. At time of birth she had two teeth on the right side of the upper jaw, just behind where the eye-teeth normally are; these were pulled by the doctor in charge a short time after birth. Her primary dentition was normal, all the teeth coming through except the one just back of the right eye-tooth. These teeth which she got when a child remained permanently, and she never had a second dentition. Her teeth were always bad, and she suffered so much from them that in 1884 she had them extracted, and has since worn artificial teeth. Ever since she can remember she has had catarrh of the right nostril, with very offensive discharge; occasionally small pieces of bone, the size of finger-nails, which were ragged and felt gritty, came away in the discharge. In 1884 she sought relief at a dispensary. At that time she could feel a gritty substance beneath her right orbit, as though there was a piece of loose bone there. She could also feel a hard substance in her nose by inserting her finger. She continued going, at more or less frequent intervals, to the dispensary. In September and October, 1895, she suffered so intensely from occipital and temporal headache that in the first week of October she went to the Out-Patient Department of the Pennsylvania Hospital, where she first came under my care. Inspection of the right nasal chamber showed a pinkish-white, glistening object on the floor of the nasal fossa, which appeared to project from it, and resembled a polypus in color. Examination by probe revealed hardness, fixation, and sensation of bone, and the object was diagnosticated a tooth, root presenting. The distance from the vestibular opening was four centimetres, the visible projecting root, one centimetre in length, directed at a slightly oblique angle towards the median line, and free from contact with the nasal chamber. The right nasal cavity was markedly irregular, the anterior portion of the lower turbinated bone being rudimentary. The surface of the floor of the nose was very irregular, with marked deflection of the nasal septum to the left. Great hyperæmia of the mucous membrane existed. The removal of the tooth was accomplished without much difficulty, by the use of long sequestrum forceps. Upon its removal the hemorrhage was free, followed some time later by secondary hemorrhage. No necrosis of the surrounding bony tissue was observed. After rapid healing a depression remained in the floor of the nose where the tooth had been. The occipital and temporal pain was markedly relieved and the local and general condition greatly improved. Examination showed the tooth to be a permanent bicuspid.

Case II.—Dr. A. W. Watson, Philadelphia, reported the following case: The case was of a man, thirty-three years of age, who had a sarcoma of the right nasal chamber, involving primarily the inferior turbinated bone, and subsequently the whole superior maxilla. During an attempt under ether to remove the diseased tissue the instrument struck a hard substance embedded in the tissues under the inferior turbinated bone; upon removal this proved to be the root, or part of the root, of a tooth.

Case III.—Hall DeHaviland reports a case of a girl, aged fifteen years, in whom there existed in the right nostril a misplaced tooth, which proved to be the right canine. There were marked signs of hereditary syphilis, and a fetid discharge issued from the nostrils. The nasal septum was perforated. The tooth was extracted from its abnormal position three months after it was first seen.

Case IV.—Schæffer, of Bremen, reports a case of a man, aged thirty-six years, who for a long time had suffered from obstruction of his left nostril. Since the age of fourteen he could feel a hard body in the left nostril with his finger. All his teeth were present in their normal position. The foreign body was extracted with a snare and forceps, and found to be an incisor, measuring in length a centimetre and a half, in its greatest breadth half a centimetre. It had for its crown enamel. On its small root it had a little cap of cartilage.

Case V.—E. Fletcher Ingals, of Chicago, in examining a patient who had for some time suffered from nasal catarrh, found on the floor of the left naris, four centimetres back from the nostril, a hard substance, feeling, when touched with a probe, like bone. On seizing it with forceps the patient suffered severe pain, like that caused by striking a decayed tooth. The pain was so intense that it was necessary to anæsthetize the patient before a thorough examination could be made. After the patient was etherized the body was engaged in a snare and drawn out, when it was found to be a supernumerary tooth, resembling closely a canine, and two centimetres in length. The tip of the root had been exposed in the nasal cavity, and below this tooth was covered with tissue which was adherent down to the crown. The dentine of the crown was perfect, excepting a small perforation at its apex, but within the tooth was decayed. The tooth must have been projecting within the nasal cavity for some time, for the portion of the apex of the root which was uncovered had lost by erosion about a millimetre in thickness from its entire circumference. He says it is comparatively rare for teeth to grow in the roof of the mouth or nasal cavities, yet several such cases have been met with. In nearly all instances the "wild tooth," as it is called by the laity, is found to be a supernumerary tooth or a misplaced canine. Professor E. S. Talbot examined the tooth and the patient's mouth, and stated from the appearance of the tartar and the green stain upon the apex of the crown it must have occupied a diagonal position in the hard palate, the crown being in the roof of the mouth, pointing towards the central incisors, and the apex of the root in the nares pointing posteriorly. A ring of tartar encircled the crown from about three millimetres below the upper surface to nine millimetres above the lower surface, and that part of the crown below the ring was covered with a fungus growth, which proved that that part only was exposed in the mouth. The tooth had decayed because of the imperfectly formed apex of the crown until the pulp had become exposed, and death had resulted, causing alveolar abscess, the sac of which came away with the tooth. He concludes, "While it is not uncommon to find supernumerary teeth in the roof and anterior part of the mouth, it is very rare to find the apex entering the nares."

Case VI.—J. S. Marshall, of Chicago, reports a case of a superior wisdom-tooth discharged from the nasal passages. A woman, aged sixty-two years, had suffered from facial neuralgia on the right side, accompanied by severe otalgia for ten years. In October, 1884, she suffered intensely from what she supposed to be a severe cold in the head. The right nostril became slightly swollen and completely obstructed. Breathing was almost impossible when the lips were closed. After an unusually great effort in coughing and blowing her nose, a few days after the onset of the cold, she suddenly felt something fall upon her tongue, which proved, upon examination, to be a large right superior wisdom-tooth, covered with fetid pus. There was immediate relief of facial pain. Thirty years previous she had all her upper teeth removed;. about four years after the removal of her teeth a tumefaction appeared upon the right superior maxilla, near the tuberosity. She consulted a dentist, but he could offer no explanation of the swelling, which disappeared in a few days. In May, 1884, she suffered from an abscess of the right ear. For several years previous to the discharge of the tooth she had had a slight fetid discharge from the nose. Dr. Marshall offers the following explanation of the symptoms, that all the pain and discomfort in the right facial region was due to this erratic tooth. The pain and swelling twenty-five years previous to the discharge of the tooth was due to the irruption of this tooth in an inverted position, which, taking a direction upward and forward, finally pierced the floor of the antrum of Highmore. The abscess in the ear must have been the result of an abscess at the roots of this tooth, which discharged its contents into the meatus, and at the same time freed the tooth from its crypt, and left it loose in the antrum. The tooth finally worked its way to the anterior portion of the antrum, and by contact with the nasal wall produced ulceration of this and the inferior turbinated bone, and thus found its way into the nasal passages. The catarrhal discharge was due to the presence of the tooth in the antrum. Dr. Marshall regards the case as interesting, in that it explains an obstinate case of trifacial neuralgia. It indicates the probable cause of a severe aural abscess.

Case VII.—The author was consulted by Mr. M., aged forty-two years, sent by a rhinologist, who stated that pus was present in the floor of the nose, and he suspected it was due to a diseased condition of the incisor teeth. Upon examination it was found that the left upper lateral had been extracted but recently, as the tooth had been very sore and the gum and surrounding tissue had been much swollen. A probe could be passed to a distance sufficiently far to reach the floor of the nose; carious bone could be detected by exploration. Absorption had taken place to such an extent that pressure on the gum tissue directly back of the tooth-socket caused pus to flow out of the opening from which the tooth had been extracted. It was found also that pus was being discharged to a limited extent into the nose, where there was a fistula. The tooth-socket was washed out with tepid water, followed by hydrogen dioxide, full strength, which distended the gum tissue on the palatine side of the socket. The carious bone was removed with a large bur, and the cavity again syringed with hydrogen dioxide and packed with carbolized gauze, to prevent gum tissue from healing from the outside rather than by granulations from within. The opening was treated for several days and responded, but did not heal until the carious bone was extensively removed, and the wound was allowed to heal from the bottom by granulation. In this case the patient was entirely relieved. The trouble in this case was caused by the abscess of the tooth producing absorption and by breaking through into the nasal cavity by line of least resistance, showing that the root of the tooth had been separated from the floor of the nose only by a thin lamina of bone or by muco-periosteum.

Case VIII.—Mr. S., aged thirty-eight years, sent by a rhinologist to see if teeth were the cause of the discharge of pus from outer wall of nose. The right superior lateral was found to be devitalized and considerably discolored. When it was proposed to open into the tooth the patient stated that he felt sure that the tooth had nothing to do with the trouble, as it had been quite recently opened and found all right. I made the remark that I could not report to his physician that the tooth was not the cause of the trouble unless I opened it. The root of the tooth had been filled with oxyphosphate or oxychloride for about two-thirds its length. The upper part of the root-canal was putrescent, and by careful examination by a fine nerve-broach, the serrations of which had been filed away, a small apical opening was found and somewhat enlarged, after which a fine exploring wire was passed for a distance of two inches by actual measurement. The probe was pushed until it met resistance of an elastic nature. As it was slightly pushed backward when the hand was removed, it showed that it had not followed the fistula, but came in contact with the periosteum and mucous membrane of the nose on the cheek side. Hydrogen dioxide was injected, but could not be forced into the nose. It was necessary, finally, to remove the tooth and bore upward a considerable distance. The opening was syringed with hydrogen dioxide, full strength, and after a few treatments a cure was effected. I have in my possession a letter written by the patient six months afterwards, stating that he had had no further nasal trouble and that the socket had entirely healed.

Case IX.—Mr. T., aged fifty-five years, was being treated for nasal catarrh, and had been using nasal syringe for two years. The patient had been having an offensive discharge from the nose for several years and never received any permanent relief, but was growing rapidly worse. I was asked by this patient, who was a person of more than ordinary medical knowledge, if it were possible for him to have nasal trouble and discharge of pus from any condition of his teeth. The patient said he asked the question as he had on the same side of the jaw as that of the nasal trouble a very badly decayed and troublesome tooth. In reply, I assured him that it was quite possible to have such a condition as he described, and explained the relation of the teeth to the antrum and of the antrum to the nose. I found, on examination, that the left superior first molar was devitalized, badly decayed, and much loosened, and was surrounded by inflamed gum-tissue, from which pus oozed on slight pressure. The cheek was much swollen, infiltrated, and painful on pressure. The patient stated that the discharge from his nose was greatest after being on his knees in church. He said that he had a dull, heavy feeling on that side of his face, which was more pronounced during cold, damp weather, and that the discharge was unilateral. All diagnostic points were in favor of abscess of antrum. The tooth was removed, and with it came away a portion of the alveolar process, necrosed and very offensive, leaving an opening into the floor of the antrum. The antrum was syringed with tepid water, at about 100° F., and afterwards, as the opening was large, with hydrogen dioxide, full strength. Here I would caution care about injecting hydrogen dioxide into any cavity, unless the opening is sufficiently large for it to escape freely after coming in contact with pus; otherwise you may give your patient considerable suffering, owing to the gas generated by the disintegrating action of the hydrogen dioxide on the pus. It is well, therefore, to first syringe the cavity with a warm alkaline solution, such as ten grains each of bicarbonate, biborate, and chloride of sodium to the ounce of water; this may be followed up by full strength hydrogen dioxide with perfect safety. The patient from first treatment had marked relief. The opening at first was closed to prevent entrance of food, or any foreign body, by packing with carbolized gauze, to which a silk ligature was tied and attached to the tooth, lest it should be drawn into the cavity by suction. This is very important, as serious trouble has in many cases been caused by pieces of cotton, plugs, tubes, etc., being allowed to enter by accident into the antrum. If such an accident should happen, the cotton might possibly be removed by enlarging the opening and engaging it by an explorer of the nature of a nerve-broach, but should a plug of any kind enter, it might be necessary to make an opening sufficiently large to explore with the finger. A case was reported to me some time since by a rhinologist, who said a patient, after weeks of suffering from antral discharge, one day sneezed, and upon examination of discharge found a piece of cotton that had been packed into an alveolar tooth-socket to stop bleeding, after the extraction of a very bad root. The piece of cotton evidently worked its way into the antrum and caused the offensive discharge, which entirely disappeared without further treatment after the cotton was sneezed out.

Dr. Brophy reports finding a piece of flexible rubber tubing used by the doctor who had treated the case unsuccessfully. Continued washing out the antrum with hydrogen dioxide and carbolized water daily for two weeks, then weekly and triweekly, as the discharge lessened, but was not able to remove the plug and cease treatment until the patient had been under treatment for about three months. The opening was kept closed by solid plug with flange attached to keep it from passing altogether into the antrum. In the flange a hole was drilled, by which means it was tied to the adjoining tooth, for fear it might drop out and get into the patient's throat.

I would state that, after considerable practical experience, drainage-tubes of whatever kind are *not a success,* for the reason that they do not drain and are difficult to keep clean.

If the tube enters at a level with the floor of the antrum, the mucous membrane will close *over* and *into* it. If it enters above the floor of the antrum, it cannot drain the bottom. If the tube has been perforated, the holes must necessarily be so small that the pus, which is generally thick and viscid, will close them up. The patient should be seen daily until the discharge is perceptibly lessened.

As regards the solid plug of silver, platinum, or hard rubber in preference to the tube, such authorities as Dr. Fillebrown, of Boston, says in an article in the INTERNATIONAL DENTAL JOURNAL, January, 1897, "I make the opening as large as a common lead-pencil, and make a rubber plug attached by a clasp to a tooth to keep the artificial canal patulous, and render the atmospheric condition normal. The plugs could easily be removed by the patient, and after cleansing be readily replaced. I have tried both the open tube and solid plugs, and much prefer the latter. It is hardly practical to use a canula large enough to syringe through freely and not to also allow the circulation of air, which is not a natural condition. The plug being easily removed, both plug and cavity can be washed thoroughly clean. A tube cannot be made clean without much trouble."

To go back to the case, I would state that an excellent result was obtained by injecting a fifty per cent. Lugol's solution, which hastened the cure. Lugol's solution is iodine, 5 parts; iodide of potassium, 10 parts, and water, 85 parts. Tincture of iodine should not be used, as it is too irritating to the mucous membrane of the sinus.

Dr. W., aged forty-eight years, sent by a rhinologist, with very offensive discharge from right upper nasal cavity. Although the patient had made careful observations, he could not account for the trouble, and was very despondent, in fact, said that he did not expect to live very long. The patient had lost all his upper teeth and was wearing a rubber plate. Upon examination an inflamed area and fistula was discovered, corresponding to position occupied by first molars. The probe came in contact with a hard substance, which was removed, and proved to be the root of a tooth. The root was embedded in the soft tissue. After extracting, the explorer was passed into the antrum, through which opening the antrum was washed with tepid water, discharging into the nose and carrying with it decomposing pus of the most offensive nature. The patient was almost entirely cured by one treatment. After three treatments the patient was discharged cured.

Burchard, of Philadelphia, reports a case of an alveolar abscess on the central incisor, with discharge from one nostril simulating a case of ozæna. This had occurred in an individual with a thin lamina of bone in the upper jaw separating it from the floor of the nose, where the inflammation had travelled by continuity of structure, by necrosis, and sinus openings into the nasal chamber. The alveolar process was unusually short; the apices of the roots of the central incisors being thereby close to the floor of the nose, inflammatory action in the nasal cavity causing change in the root of the tooth, from which it received its blood-supply, would bring about rapid pathological changes in structure. Burchard calls attention to the fact that abscesses penetrating into the nose following the line of least resistance are due to the presence of the anterior palatine and incisor foramina, which are situated immediately behind the incisor teeth.

Irregularities and abnormalities as to formation of the accessory cavities, the antrum, etc., may explain many of the peculiar or unique cases often reported. Cryer has shown by his sections of the skull, with a view to demonstration of the relation of accessory cavities to the nasal chamber, that almost any size or shape of cavity or thickness of bone is possible, the antrum cavities varying in size from a little larger than a pea to three times the usual size, and extending under the floor of the nose. In the cases associated with nasal lesion, it is quite likely that the chronic inflammatory process set up in the floor of the nose may interfere with the nervous and vascular supply of the tooth directly under it, causing atrophic function with devitalization.

Irregular nasal breathing will affect the regular development of the upper jaw and facial bones, not including the lower maxilla. It will be noted in children with nasal obstruction that the upper and lower jaws do not fit, due to the fact of arrested or irregular development in the upper portion of the face, with no interference to the lower maxilla. The peculiar staring countenance of mouth-breathers includes more than the mere unnatural expression of the face produced by the constant wide-open mouth. The masseter muscles, instead of being developed and trained to hold the jaw in its proper place, are developed and trained to allow the mouth to remain open, and even after the removal of nasal obstruction in childhood, it requires weeks of training to get the child to keep the mouth closed, although it will be found on closure of the mouth perfect nasal breathing has been established. It is usually attributed to force of habit; but in reality the muscles are not accustomed to holding the jaw in that position, and it is maldevelopment.

Hubbard, of New York, on "Immediate and Remote Causes of Malformation of the Dental Arch," in a paper read before the First District Dental Society of the State of New York, December 14, 1897, as to what is a normal upper arch, said "that which is most useful and performs its functions most perfectly, according to the environments in which it is found, may be useful and perform its function properly, and still not be a thing of beauty. In many cases irregularity of the upper arch allows the person to perform the function of mastication, in fact all the functions performed by the teeth, and yet the relation of the upper and lower jaw is not a perfect one. The period after birth up to the time of the osseous union which occurs in the facial bones is the time to correct irregularities; after that time it is almost impossible to alter the shape of the upper jaw or the nasal cavities, except by removal of the bony structure. Kölliker was the first to call attention to the fact that in whatever direction the jaw has increased, the increase has been produced by additions to the external surface. Obstructive lesions of the pharynx, naso-pharynx, and the nose in childhood then must play an important relation to facial development. Possibly the most important of all is deflection of the septum and enlargement of the pharyngeal tonsils. There is a constitutional or inherent tendency in children of a lymphatic temperament to enlargement of the lymphoid structure. This must be taken into consideration from a stand-point of prognosis. Pressure on the face at birth may have considerable to do with the starting of facial irregularities, or the careless placing of a child in the same position, allowing pressure laterally or antero-posteriorly to be kept up in the same line for days and weeks, may be the starting-point of facial irregularities. The proof of the vastly important relation of nasal breathing to irregularities of the teeth is shown by this fact, that, in a great majority of cases with irregular teeth, whether it be in childhood or in adult life, the history will show obstruction within the nasal cavity at some early period in the individual's life. It may be in an adult, and the lymphoid structure in the naso-pharynx has entirely atrophied and disappeared, but the marks of that interference in respiration is evident in the development of the upper face and the upper jaw; the two are intimately associated.

The effect of thumb-sucking on the development of the floor of the nose and the anterior part of the superior maxilla is almost characteristic. While this deformity or malformation is not brought about by obstruction to nasal breathing, yet it shows the marked influence which suction or pressure will have on the soft developing facial bones.

